# Association of working conditions including digital technology use and systemic inflammation among employees: study protocol for a systematic review

**DOI:** 10.1186/s13643-020-01463-x

**Published:** 2020-09-28

**Authors:** Helena C. Kaltenegger, Linda Becker, Nicolas Rohleder, Dennis Nowak, Matthias Weigl

**Affiliations:** 1 Institute and Clinic for Occupational, Social and Environmental Medicine, University Hospital, Ludwig-Maximilians-University of Munich, Ziemssenstraße 1, 80336 München, Germany; 2 Chair of Health Psychology, Institute of Psychology, Friedrich-Alexander University Erlangen-Nürnberg, Erlangen, Germany

**Keywords:** Work, Job, Health, Working conditions, Occupational stress, Digitalization, Technostress, Inflammation, Inflammatory markers, Immune system

## Abstract

**Background:**

With the dynamic advancement of digitalization, working environments are changing and risk for employee stress may be increasing. Work stress has been associated with a dysregulation of inflammatory processes as a component of immune function. Systemic low-grade inflammation is discussed as a key player in the relation between stress exposure and chronic illness, such as cardiovascular diseases. The objective of this investigation will be to evaluate the association of working conditions including digital technology use and systemic inflammation among employees.

**Methods:**

We designed and registered a study protocol for a systematic review of randomized controlled trials and prospective non-randomized studies (e.g., cohort, interrupted time series, or before-after studies). We will include studies conducted among adult workers reporting associations of working conditions and inflammatory activity. The outcome will be biomarkers of systemic low-grade inflammation on cell, plasma molecule and intracellular level, such as C-reactive protein, or different types of leukocytes, cytokines, etc. Literature searches will be conducted in several electronic databases (from January 1982 onwards), including PubMed/MEDLINE, Embase, PsycINFO, Web of Science, and CENTRAL. Two reviewers will independently screen all retrieved records, full-text articles, and extract data. The study methodological quality (or bias) will be appraised using appropriate tools. Our results will be described qualitatively. Random effects meta-analysis will be conducted, if feasible and appropriate. Additional analyses will be performed to explore potential sources of heterogeneity.

**Discussion:**

This systematic review and meta-analysis will provide a synthesis of studies evaluating the association of working conditions and systemic inflammation. We anticipate our findings to identify knowledge gaps in the literature that future research should address. Moreover, results of our review may provide implications for corporate and public policy action for employee health promotion and prevention of occupational stress.

**Systematic review registration:**

PROSPERO ID: CRD42020166887

## Background

In the light of contemporary and profound changes in the world of work coined by digitalization, the workplace may represent a significant cause of stress, and employee health promotion becomes increasingly important. Exposure to work stress has been associated with physical and mental ill health, such as cardiovascular diseases, type 2 diabetes, and clinical depression, as well as mortality [1–6]. With the dynamic advance of technologization and digitalization in humans’ workplaces and private lives, stress experience related to the interaction with information (and communication) technologies and systems has become a phenomenon of rapidly growing scholarly interest. Research on *technostress* [7] and *digital stress* [8–11] has identified a broad range of potential sources of stress, such as techno-invasion, interruptions, information overload, complexity, invasion of privacy, etc. [12–15], and moreover, has revealed effects on the endocrine system with possible implications for immune function [16].

Sustained low-grade inflammation as a sub-component of the immune system is discussed as a central process in the association between stress exposure and severe long-term diseases [17–20]. Inflammation is mostly assessed by measuring levels of inflammatory markers in plasma or serum, including (proinflammatory) cytokines, such as interleukins (IL; mainly IL-1 (β) and IL-6), tumor necrosis factors (TNF; mainly TNF-α), interferons (IFN), and the acute-phase protein C-reactive protein (CRP) [21–23]. Several studies have shown associations between adverse working conditions, such as high effort-reward imbalance [24], organizational injustice or shift work, and inflammatory activity [25–27]. Reviews and meta-analyses indicate substantial evidence for the relation of acute and chronic psychosocial stress with immune function and inflammatory processes [17, 21, 28, 29]. However, there is a paucity of reviews and meta-analyses examining systemic inflammatory processes due to working conditions and/or work-related stress. Previous reviews and meta-analyses have focused on associations between workplace stress and immune function [30, 31], and on work-related psychosocial factors and inflammatory markers [32]. However, to our knowledge, there are no systematic reviews and meta-analyses investigating the strength of the evidence on the prospective association of various modern working conditions including digital technology use and employees’ systemic inflammatory markers.

We plan to conduct a systematic review on the association of working conditions including digital technology use and systemic inflammation among employees. There are two aims within the planned review: The first aim will be to determine the current evidence on associations between working conditions and inflammation based on randomized controlled trials and prospective non-randomized studies and to assess if different classes of working conditions are differently associated with inflammatory markers. The second aim will be to identify if there are studies specifically investigating work-related use of digital technologies and inflammatory markers, and if so, evaluate their potential associations.

## Methods

### Protocol registration and reporting information

The present protocol has been registered within the PROSPERO database (registration ID: CRD42020166887). This protocol is being reported in accordance with the reporting guidance provided in the Preferred Reporting Items for Systematic Review and Meta-analysis Protocols (PRISMA-P) statement [33, 34] (see PRISMA-P checklist in Additional file 1). The proposed systematic review will be reported in accordance with the reporting guidance provided in the Preferred Reporting Items for Systematic Reviews and Meta-analyses (PRISMA) statement [35, 36]. Information on roles, responsibilities, and skills of review team members is provided in Additional file 2.

### Eligibility criteria

Studies will be selected according to the following study characteristics: study design, participants, exposure/interventions, comparators, outcomes (PECO/PICO framework):

- *Study design*: Eligible studies will be prospective field studies reporting associations between working conditions and systemic inflammation. We will include observational and interventional studies, i.e., randomized controlled trials and non-randomized studies of interventions, such as controlled before-and-after studies or interrupted time series designs. There will be no restrictions by type of occupational setting. Laboratory studies will be excluded.
- *Participants:* We will include all types of adult workers and employee samples. We will consider all types of occupations, such as managers, professionals, technicians and associate professionals, clerical support workers, services and sales workers, agricultural workers, craft and related trades workers, plant and machine operators and assemblers, and elementary occupations (see [37]); as well as sectors, such as agriculture/forestry, chemical industries, commerce, construction, education, engineering, financial services, health services, transport, etc. (see [38]). Unemployed individuals, students, athletes, artists, military personnel, and clinical samples based on specific diagnoses will be excluded.
- *Exposures/interventions*: Studies assessing all kinds of working conditions will be eligible, encompassing a broad range of work- and employment-related aspects, like working time, compensation, mental and physical demands [39]. We will include studies examining all kinds of psychosocial factors at work, such as work environment, job content, and organizational conditions [40]. Moreover, studies focusing on work-related use of digital technologies and media as well as associated stressors, such as techno-overload or interruptions [15, 41], will be included. We define digital technologies as the entirety of all electronic devices (hardware) and applications (software) that use information based on number codes, and the entirety of all media coded in formats that can be processed by these devices and applications. Work-related digital technologies may comprise computers, e-mails, mobile phones, internet, messaging systems, artificial intelligence, autonomous systems, robots, virtual reality, etc. Studies examining specific environmental hazards, i.e., chemical or physical agents in the air, water, soil, food, or extreme heat, will be excluded. We will exclude studies assessing shiftwork (for a review, see, e.g., [42]) and socioeconomic status as exposure variables. Studies investigating all kinds of workplace-related interventions, that is, all measures directly or indirectly aiming at occupational health promotion (on or off site), will be eligible. We will exclude studies on nutraceutical interventions.
- *Comparators:* If applicable, the comparator group will be based on subjects not exposed to a specific working condition/exposed to a lesser extent or not exposed to a specific workplace intervention/exposed to a different intervention.
- *Outcomes*: Our outcome of interest will be changes in markers of systemic low-grade inflammation measured in the blood or saliva. We will consider three main categories of indicators of systemic inflammation: cells, plasma molecules, and intracellular processes. Regarding cells, we will include studies examining leukocytes (and subtypes) and dendritic cells. With regard to plasma molecules, we will include studies on the acute-phase proteins CRP, fibrinogen, and serum amyloid A, and on cytokines including different chemokines, interleukins, lymphokines, and monokines as well as IFN-γ and TNF-α. Cell-free DNA, inflammasomes, and intercellular adhesion molecule-1 will also be included as target outcomes. Concerning intracellular processes, we will include studies on the transcription factors AP-1, NF-IL6, and NF-kappa B, and on gene expression associated with inflammatory processes (see Table 1 for a list of included markers). We will exclude studies assessing inflammatory markers as indicators of organ damage, such as kidney injury. All inflammatory markers will be considered as main outcomes; there will be no prioritization or secondary outcomes. We will include studies with at least one follow-up measure, i.e., two consecutive measurements, of outcome variables.

**Table 1:** Outcome category, definition, and included inflammatory markers per category

Outcome category	Definition of outcome category	Inflammatory markers (per outcome category)
Cells	Inflammation-related processes on cell level as a component of cellular immunity	Leukocytes Eosinophils Granulocytes Lymphocytes Macrophages Monocytes Neutrophils Dendritic cells
Plasma molecules	Inflammation-related processes on plasma protein level as a component of humoral immunity	Acute-phase proteins C-reactive protein (CRP) Fibrinogen Serum amyloid A
		Cytokines Chemokines Interferon-gamma (IFN-γ) Interleukins (IL) Lymphokines Monokines Tumor necrosis factor-alpha (TNF-α)
		Cell-free DNA
		Inflammasomes
		Intercellular adhesion molecule-1
Intracellular processes	Inflammation-related processes on intracellular level	Transcription factors AP-1 NF-IL6 NF-kappa B Gene expression Transcripts for proteins associated with inflammatory processes Transcriptomics focusing on or revealing inflammatory processes

We will include studies published from January 1982 (considering the introduction of the term *technostress* [7] in 1982) onwards. We will include articles in peer-reviewed journals reported in the languages English and German. A list of possibly relevant titles in other languages will be provided in the final study report as an appendix. We will exclude conference proceedings, dissertations, or theses.

### Information sources and search strategy

The primary source of relevant literature will be a structured search using several electronic databases (from 1982 onwards): PubMed/MEDLINE, Embase, PsycINFO, Web of Science, and Cochrane Central Register of Controlled Trials (CENTRAL). The secondary source of potentially relevant material will be a search for the difficult to locate literature, including Google Scholar. We will perform hand searching of reference lists of included studies and relevant reviews to identify additional eligible papers. Experts and prolific authors in the field will be contacted and consulted. The literature searches will be designed and conducted by the review team. The search will include a broad range of terms and keywords related to the PECO/PICO question (e.g., “worker”, “job control”, “social support”, “communication technology”, “digital stress”, “inflammation”, “immune system”). A full draft of the search strategy for PubMed/MEDLINE is provided in Additional file 3. This search strategy will be adapted to the other databases using the software Systematic Review Accelerator [43].

### Screening and selection procedure

All retrieved titles and abstracts of identified articles will be imported into the software *EndNote* X8 (Thomson Reuters). The screening process will be conducted using the web and mobile application Rayyan [44]. Two independent reviewers (HK, MW) will conduct a systematic and stepwise selection of eligible studies, that is, screening of titles, abstracts, and full texts. Reviewers will discuss potential discrepancies until a consensus is reached. Potential conflicts between the two reviewers will be resolved after the consultation of a third reviewer (LB) from the study team. Excluded studies will be recorded. All steps of the study selection will be tested prior, in order to identify potential misunderstandings between the reviewers regarding the eligibility criteria or the software interface. This pre-test will include a random sample of 20 records. A flow diagram presenting the study selection process will be prepared [35].

#### Data collection process

Data will be extracted independently by two authors (HK, MW) and imported into *Excel* (Microsoft Office Professional Plus, 2016). This step will be pre-tested with five articles to test for feasibility and comprehensiveness. A third reviewer (NR) from the study team will be included as a consultant in case of disagreement. Several main categories and individual data will be extracted from all eligible articles (see Table 2). In the case of missing information, corresponding authors will be contacted to obtain information relevant to this review. If there are multiple reports of a single study, only the key paper will be included (authors will be contacted, if not clear).

**Table 2:** Main categories and data extracted from included articles

Main categories		Data to be extracted
I	Study characteristics	- Authors and year of publication
		- Study design
		- Country of study
		- Period of follow-up and follow-up rate
		- Occupational setting
II	Samples	- Participants: demographics, professional characteristics, health-related variables
		- Sample size
III	Type and assessment of exposures/interventions and comparators	- Type of working condition (e.g., job demands, job control, workload, social support, digital technology use)
		- Type of workplace intervention (e.g., physical activity, stress reduction)
		- Type of comparator
		- Methods of assessment
IV	Type and assessment of outcomes	- Category and type of inflammatory markers
		- Source of outcomes (blood, saliva)
		- Method/technique of assessment
V	Statistical analyses and reported results	- Type of statistical methods and analyses
		- Means and variance metrics of inflammatory markers (e.g., standard deviation, confidence intervals)
		- Coefficients (β, γ) and/or measures of strength of associations between working conditions and inflammatory markers (OR, RR, HR with SE, and/or 95% CI)
		- Effect sizes (if reported or calculable)
		- *P* -values
VI	Moderators/control of confounders	- Potential moderator or confounder variables or analyses (if reported)
		- Results of respective analyses (if reported)
VII	Further study information	- Further information of potential interest (e.g., limitations, restrictions to validity)

### Data processing and classification of exposure and outcome variables

**Fig. 1:**
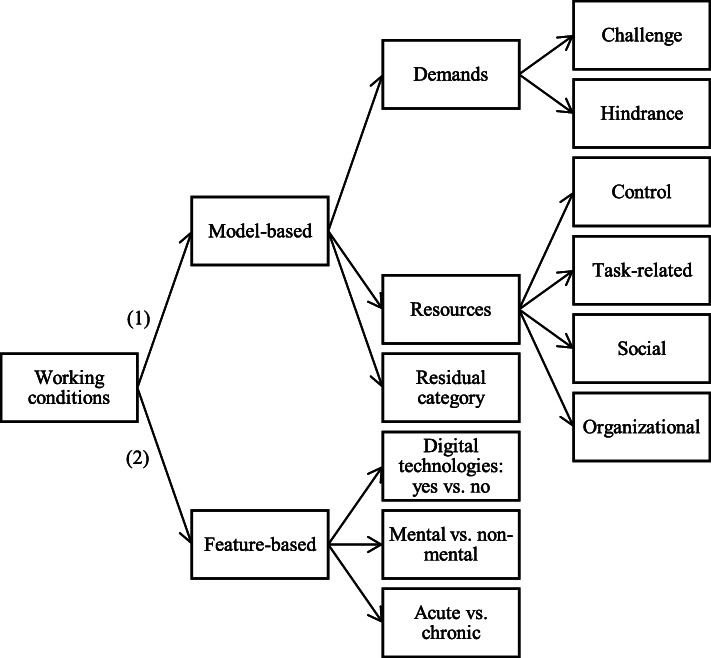
Criteria-based algorithm for model- and feature-based classification of working conditions reported in eligible studies

With regard to the exposure variables, two reviewers (HK, MW) will perform a criteria-based classification of working conditions according to the scheme presented in Fig. 1. First, included studies will be categorized concerning their underlying theoretical model. Working conditions will be classified into job demands and resources as main categories with respective subcategories based on the following four models:

- *Job demand-control model* (JDC [45]): The JDC model postulates that mental strain in a workplace context results from the combination of the two dimensions job demands and job control. High job demands, such as time pressure or workload, and low decision latitude are associated with mental strain and job dissatisfaction.
- *Job demand-control-support model* (JDCS [46, 47]): This model is an extension of the JDC model by integrating the dimension social support.
- *Job demands-resources model* (JDR [48]): The JDR model suggests that high job demands lead to strain and health impairment, whereas high resources lead to increased motivation and productivity [49]. In the long-term, an interaction between extreme job demands, which lead to exhaustion and a lack of resources leading to disengagement from work, can result in the development of burnout [48].
- *Challenge-hindrance stress model* (C-H [50]). The C-H stress model proposes that work stressors can be divided into two categories (challenge vs. hindrance stressors), which are differently associated (positively vs. negatively) with work outcomes (see [51, 52]). In the primary investigation, challenge stressors—related to the phenomenon of eustress—were shown to be positively associated with job satisfaction and negatively with job search, in contrast to distress evoking hindrance stressors found to be oppositely associated with these outcomes [50].

We are aware that hybrid models do exist in this context, such as the differentiated JDR model [53, 54], and we will classify studies accordingly, i.e., multiple allocations are possible. With regards to the category “resources”, in line with the JDR model [48], we will focus on external resources since they are more likely to be subjected to job design approaches in contrast to rather stable internal resources, such as cognitive features (see [55]); we will further distinguish between control, task-related, organizational, and social resources. If working conditions in eligible publications are reported without an underlying model, these studies will be assigned to a residual category. In addition, we will conduct a feature-based classification of every study concerning specific characteristics of the work situation irrespective of the underlying model. First, we will identify if working conditions involve digital technology use or not (as defined above). Second, we will distinguish between mental vs. non-mental, i.e., intellectual vs. physical, working conditions. Third, we will consider the time frame of working conditions, i.e., we will differentiate between acute and chronic (provided by author definition) working conditions (see Fig. 1).

### Risk of bias in individual studies

To evaluate the methodological quality of all eligible studies as well as potential limitations to validity, a standardized risk of bias assessment will be performed. Since this review will include investigations using different study designs, three different established tools to assess the risk of bias will be applied by two independent reviewers (HK, MW). (1) For randomized controlled trials, the *Cochrane Risk of Bias Tool* (RoB 2 [56]) will be used. (2) For non-randomized studies of interventions, the *Risk Of Bias In Non-randomized Studies – of Interventions* (ROBINS-I [57, 58]) tool will be utilized. These two risk of bias assessment tools include sets of questions addressing different domains of potential sources of bias from selection to reporting, and to be answered with proposed judgements. (3) For prospective observational studies, the checklist *Quality of Reporting of Observational Longitudinal Research* [59] will be applied. In case of disagreement between both reviewers, a third reviewer (LB) from the study team will be consulted and act as a tiebreaker to obtain a final evaluation. Our risk of bias assessment will be pre-tested using a sample of five, randomly selected articles of each study design category (see above). This step will ensure a joint understanding and application of the risk of bias evaluation tools between all reviewers.

### Data synthesis

First, we will provide a qualitative summary of the information extracted from each included study and of our risk of bias assessment in narrative and tabular form. In case of substantial heterogeneity and inappropriateness of statistical pooling, we will apply graphical summary approaches for evidence synthesis in the absence of meta-analysis, i.e., harvest plots, effect directions, or bubble plots for summarizing information in an accessible and user-friendly manner (see [60]).

Secondly, if a sufficient number of high-quality studies with a relatively low level of heterogeneity is retrieved, we will quantitatively synthesize data from primary studies in a meta-analysis, using *R* 3.5.2 (package: *metafor* [61]). Due to anticipated heterogeneity of effects in individual studies, we will select a random effects model based on the DerSimonian and Laird [62] method, in order to estimate the average of the effects across studies. Heterogeneity will be assessed by estimating the variance between primary studies using Cochran’s *Q* test [63] and *I*^2^ statistic [64]. Results will be illustrated graphically using forest plots including individual study effects (step 1) and combined effect estimates (step 2) as well as confidence intervals, respectively [65].

#### Additional analyses

If the number of identified studies allows for, potential sources of heterogeneity will be explored further by subgroup analyses or meta-regression based on PECO/PICO and study design characteristics [66, 67]. We intend to perform subgroup analyses for potential effect modifications by age (e.g., young vs. middle-aged vs. elderly professionals) and sex (men vs. women), as age and sex are important determinants in work-related stress level, yet with inconsistent effects reported in the literature [68, 69]. Moreover, we plan to group studies regarding the type of exposure according to our criteria-based classification of working conditions (model based: demands vs. resources; feature based: digital vs. non-digital, mental vs. non-mental, acute vs. chronic). We will distinguish between observational and interventional study designs, and if enough studies will be identified, we intend to provide post hoc classifications regarding workplace interventions (e.g., organizational/structural vs. individual/behavioral). In case a sufficient set of studies with a large variety of outcomes is retrieved, subgroup analyses based on our defined outcome categories (i.e., cells, plasma molecules, intracellular processes; see Table 1) will be undertaken. Finally, we plan to conduct sensitivity analyses to test for effects of exclusion of particular studies on the results based on methodological quality according to the risk of bias assessment and measurement/source of inflammatory markers (i.e., exclusion of studies using salivary markers) [67].

#### Meta-biases

The results of the review and meta-analysis will be critically examined with respect to sources of meta-bias, such as selective reporting within studies or publication bias across studies. We plan to generate a funnel plot, and tests for asymmetry (e.g., Egger’s test [70]) including at least 10 studies (if possible) will be performed to check for small-study effects [67, 71, 72]. Furthermore, we will apply the critical appraisal tool for systematic reviews on randomized and/or non-randomized studies of healthcare interventions AMSTAR-2 [73].

#### Confidence in cumulative evidence

The strength of the body of evidence will be assessed by using the Grading of Recommendations Assessment, Development and Evaluation (GRADE), a system for rating quality of evidence and strength of recommendations [74, 75]. Quality of evidence refers to the confidence that the estimates of the effect are correct and can be classified in one of four levels—high (“further research is very unlikely to change our confidence in the estimate of effect”), moderate (“further research is likely to have an important impact on our confidence in the estimate of effect and may change the estimate”), low (“further research is very likely to have an important impact on our confidence in the estimate of effect and is likely to change the estimate”), and very low (“any estimate of effect is very uncertain”) [74, 76]. Threats to quality of evidence comprise study limitations, inconsistency of results, indirectness of evidence, imprecision as well as publication bias, and quality rating can be downgraded by the presence of these five factors [77]. The strength of recommendation is defined as the confidence that the desirable effects of an intervention outweigh its undesirable effects and can be graded as strong or weak [74, 78]. GRADE has been successfully used in the fields of clinical medicine, public health, and policy making, and more recently, its application has been advanced to studies in occupational and environmental health [79, 80].

## Discussion

This protocol describes the methodology for a systematic review of the current study base on the association between working conditions including digital technology use and inflammatory markers in employees. We propose a rigorous assessment and synthesis of the current literature base with particular focus to high-quality studies, potentially allowing for evidence-based inferences concerning the actual state of knowledge on employees’ inflammatory level that can be attributed to working conditions and job environment.

The protocol specifies all necessary steps of our systematic review limiting any potential bias that may occur a posteriori. It includes well-defined criteria for searching the scientific literature, study selection, risk of bias assessment, data extraction, and synthesis of findings. However, we are aware of potential sources of bias that may exist a priori and are foreseeable. Since our search does not include unpublished data and gray literature, we acknowledge that selection bias in terms of publication bias may occur. Moreover, language bias may be introduced by restrictions of eligible publications to English and German language. Although we cannot exclude that relevant studies have been conducted and/or published in other languages, we are confident that the validity and precision of our findings will not be substantially affected by language bias. Previous research suggests that restrictions beyond the English language introduce little to no systematic bias [81–83].

In the light of the limitations of previous and current research attempts [30–32], our investigation aims at advancing knowledge on the association of working conditions and systemic inflammation among employees particularly by three contributions: (1) We will only incorporate prospective studies to draw our effect estimates upon on a study base with high level of evidence designs. (2) A criteria-based classification of working conditions based on prominent job stress models will be provided, and digital technology use will be considered as a particular type of exposure. (3) Our review will include a comprehensive set of inflammatory markers, encompassing phenomena on different molecular biological levels (i.e., cells, plasma molecules, intracellular processes) and taking into account recent advancements in stress biomarker research, such as cell-free DNA. This broader scope expands the set of potential markers that indicate dysregulation of inflammatory processes associated with working conditions and will allow inferences concerning the feasibility and utility of bio-psychological markers for the assessment of employees’ inflammation level in occupational contexts.

Difficulties in the procedure of our planned review could relate to the retrieval of a large amount of records not relevant to our research question, due to the extensive set of search terms. An important challenge might be the rigorous and consistent exclusion of non-eligible studies according to our PECO/PICO characteristics. For instance, we anticipate that many of the retrieved studies include samples with previous medical conditions, such as cardiovascular diseases, or address inflammation as an acute clinical condition (e.g., in connection with injury) rather than chronic subclinical inflammation. Any amendments made to the protocol will be depicted and documented in the final publication of the review. The completed review is intended to be published in a peer-reviewed journal in the field of occupational medicine or work psychology and will be presented at scientific conferences and other scientific outlets.

Several limitations of our anticipated investigation have to be considered on individual study and review level. Although our classification of working conditions is based on well-established occupational stress models, the selection may be regarded as arbitrary since other theories and models of great importance do exist in this field (e.g., effort-reward imbalance model [24]). Moreover, the validity and key propositions of the included models remain subject to scientific scrutiny: While considerable support for additive effects of job demands, job control and social support on psychological well-being (“strain hypothesis”, “iso-strain hypothesis”) has been found, evidence on moderating effects of job control or social support (“buffer hypothesis”) is less consistent, and support in longitudinal compared with cross-sectional studies appears to be weaker [84, 85]. Concerning the C-H model, negative associations with key variables, such as psychological strains or physical health, have been demonstrated for both types of stressors in a recent meta-analysis, confining applicability of the model to few outcomes and questioning the alleged beneficial role of challenge stressors [86]. Nevertheless, keeping these limitations in mind, we assume that our suggested algorithm provides a sensible and feasible categorization of all sorts of studied working conditions. With regard to digital working conditions, we acknowledge that possible applications of digital technologies are multimodal and workplace settings significantly differ in their utilization of tools and modes of digital communication and information technologies, what may limit comparisons across professional settings and samples [87]. Furthermore, we anticipate potential limitations concerning the validity of the outcome inflammation. The selection of inflammatory markers often depends on aspects of measurability and feasibility rather than their pure indicative value for inflammation. Hence, we expect to detect a large body of research on CRP measurements, but, at the same time, we aim to capture other useful parameters of chronic low-grade inflammation by including a broad range of markers previously identified in stress research in our search strategy. With respect to the assessment method of inflammatory markers, we are aware of potential confinements regarding salivary markers (see [17, 88]) and we will consider the assessment method in the data analysis (see above). In addition, inflammation involves a complex interplay of biological processes and should not be assessed in isolation underlining the necessity of adjusting for other variables, such as general health, including physical and mental health conditions associated with inflammation as well as anthropometric parameters, most importantly body fat percentage (or BMI, WHR, etc.). Lastly, the generalizability of the findings of our review will be limited to the working population.

Given the increasing interest and need for knowledge on the effects of modern working conditions on employee health and well-being, we regard our research question as highly relevant. Although we are aware of the difficulties concerning the feasibility of studies with rigor designs and high-level methodology in occupational settings (i.e., randomized controlled trials), we assume that our review results will inform future research in this field in several ways. First, our findings will reveal recommendations for the conductance of high-level investigations in occupational practice settings. Second, the findings will provide guidance for future studies on approaches to improve working conditions with the objective to promote employee health and well-being. Third, this review may help to identify research gaps concerning the effects of specific, but important working conditions, such as those shaped by recent societal developments. Eventually, as a practical implication, collated evidence on the effects of workplace interventions on systemic inflammation may yield indications for corporate and public policy action on employee health promotion.

## Supplementary information

**Additional file 1.** PRISMA-P checklist (file format: pdf)

**Additional file 2.** Review team roles and responsibilities (file format: pdf)

**Additional file 3.** Search strategy for PubMed/MEDLINE (file format: pdf)

## Data Availability

Not applicable.

## Abbreviations

IL: Interleukin
TNF: Tumor necrosis factor
IFN: Interferon
CRP: C-reactive protein
AP-1: Activator protein 1
NF-IL6: Nuclear factor for interleukin-6 expression
NF-kappa B: Nuclear factor “kappa-light-chain-enhancer” of activated B cells
JDC: Job demand-control model
JDCS: Job demand-control-support model
JDR: Job demands-resources model
C-H: Challenge-hindrance stress model
BMI: Body mass index
WHR: Waist-to-hip ratio
