# Supplementary material for: Association of working conditions including digital technology use and systemic inflammation among employees: study protocol for a systematic review
Source: Syst Rev. 2020 Sep 28;9:221. doi: 10.1186/s13643-020-01463-x (PMC7523305; doi:10.1186/s13643-020-01463-x)
Supplement: Supplementary file 2 — Additional file 2. Review team roles and responsibilities (file format: pdf) [file 13643_2020_1463_MOESM2_ESM.pdf]

Table A1

Team members of the planned systematic review and meta-analysis, their roles, applicable knowledge and skills as well as respective responsibilities

**Team member**	**Applicable knowledge and skills**	**Responsibilities**
Helena Kaltenegger	Psychological risk factors for mental and physical health, health and well-being in the workplace	Development of search strategy, study selection, data extraction, data processing and classification of variables, risk of bias assessment, data synthesis, content drafting and approval
Linda Becker	Stress physiology, psychological risk factors for mental and physical health, systematic reviews and meta-analysis	Stress physiology consultation, systematic review methods consultation, evidence evaluation, content review and approval
Nicolas Rohleder	Stress physiology, immune function and inflammation, stress system regulation of inflammatory processes, psychoneuroimmunology, systematic reviews and meta-analysis	Psychoneuroimmunology consultation, systematic review methods consultation, evidence evaluation, content review and approval
Dennis Nowak	Occupational and internal medicine, workplace risk factors for health, industrial safety, systematic reviews and meta-analysis	Medical physiology and systemic inflammation consultation, evidence evaluation, content review and approval
Matthias Weigl	Psychosocial risks at work, Well-being outcomes at work, occupational stress, Work analysis, systematic reviews, occupational epidemiology	Systematic review methods consultation, study selection, data extraction, data processing and classification of variables, risk of bias assessment, evidence evaluation, content review and approval
