# Supplementary material for: Association of working conditions including digital technology use and systemic inflammation among employees: study protocol for a systematic review
Source: Syst Rev. 2020 Sep 28;9:221. doi: 10.1186/s13643-020-01463-x (PMC7523305; doi:10.1186/s13643-020-01463-x)
Supplement: Supplementary file 3 — Additional file 3. Search strategy for PubMed/MEDLINE (file format: pdf) [file 13643_2020_1463_MOESM3_ESM.pdf]

Database	Boolean operator	Domain according to PICO/PECO and study design
		**Participants**
PubMed		“work”[tw] OR worker*[tw] OR working[tw] OR works*[tw] OR worka*[tw] OR employ*[tw] OR manager*[tw] OR colleague*[tw] OR coworker*[tw] OR occupation*[tw] OR company[tw] OR companies[tw] OR office*[tw] OR business*[tw] OR job[tw] OR organization*[tw] OR organisation*[tw] OR industr*[tw] OR “workplace”[MeSH Terms] OR workplace*[tw] OR professionals[tw] OR profession[tw] OR professions[tw] OR professional[tw]
		**Intervention/ exposure and comparators**
	AND	job demand*[Title/Abstract] OR work demand*[Title/Abstract] OR high demand*[Title/Abstract] OR job control[Title/Abstract] OR work control[Title/Abstract] OR low control[Title/Abstract] OR lack of control[Title/Abstract] OR decision latitude[Title/Abstract] OR decision authority[Title/Abstract] OR skill level*[Title/Abstract] OR decision-making freedom[Title/Abstract] OR intellectual discretion[Title/Abstract] OR skill discretion*[Title/Abstract] OR workload*[Title/Abstract] OR work load*[Title/Abstract] OR job strain[Title/Abstract] OR work strain[Title/Abstract] OR responsibility[Title/Abstract] OR responsibilities[Title/Abstract] OR autonomy[Title/Abstract] OR task organization[Title/Abstract] OR task organisation[Title/Abstract] OR skill variety[Title/Abstract] OR time pacing[Title/Abstract] OR decision resource*[Title/Abstract] OR repetitious[Title/Abstract] OR repetitive[Title/Abstract] OR monotonous[Title/Abstract] OR creative[Title/Abstract] OR creativity[Title/Abstract] OR work-place conflict*[Title/Abstract] OR excessive[Title/Abstract] OR hectic[Title/Abstract] OR psychologically demanding[Title/Abstract] OR “job satisfaction”[MeSH Terms] OR job dissatisfaction[Title/Abstract] OR work satisfaction[Title/Abstract] OR work dissatisfaction[Title/Abstract] OR work overload*[Title/Abstract] OR work over-load*[Title/Abstract] OR “social support”[MeSH Terms] OR social support[Title/Abstract] OR support system*[Title/Abstract] OR social network*[Title/Abstract] OR emotional support[Title/Abstract] OR cooperation[Title/Abstract] OR collaboration[Title/Abstract] OR social isolation[Title/Abstract] OR collective[Title/Abstract] OR task variety[Title/Abstract] OR planning of work[Title/Abstract] OR education[Title/Abstract] OR social interaction[Title/Abstract] OR social contact*[Title/Abstract] OR client contact[Title/Abstract] OR customer contact[Title/Abstract] OR high strain[Title/Abstract] OR low strain[Title/Abstract] OR time pressure[Title/Abstract] OR under-stimulation[Title/Abstract] OR understimulation[Title/Abstract] OR underload[Title/Abstract] OR under-load[Title/Abstract] OR psychosocial[Title/Abstract] OR “interpersonal relations”[MeSH Terms] OR job resource[Title/Abstract] OR job

	resources[Title/Abstract] OR recipient contact[Title/Abstract] OR physical demand*[Title/Abstract] OR physically demanding[Title/Abstract] OR shift work*[Title/Abstract] OR work shift*[Title/Abstract] OR feedback[Title/Abstract] OR reward[Title/Abstract] OR rewards[Title/Abstract] OR participation[Title/Abstract] OR job security[Title/Abstract] OR job insecurity[Title/Abstract] OR supervisor support[Title/Abstract] OR "Stress, Psychological"[MeSH Terms] OR stressor[Title/Abstract] OR stressors[Title/Abstract] OR stressful[Title/Abstract] OR noise[Title/Abstract] OR heat[Title/Abstract] OR qualification[Title/Abstract] OR overtaxing[Title/Abstract] OR challenge[Title/Abstract] OR challenges[Title/Abstract] OR challenging[Title/Abstract] OR challenge-related[Title/Abstract] OR hindrance[Title/Abstract] OR hindrances[Title/Abstract] OR hindrance-related[Title/Abstract] OR organizational commitment[Title/Abstract] OR organisational commitment[Title/Abstract] OR distress[Title/Abstract] OR di-stress[Title/Abstract] OR disstress[Title/Abstract] OR eustress[Title/Abstract] OR eu-stress[Title/Abstract] OR organizational politics[Title/Abstract] OR organisational politics[Title/Abstract] OR red tape[Title/Abstract] OR "staff development"[MeSH Terms] OR effort[Title/Abstract] OR efforts[Title/Abstract] OR effort-reward*[Title/Abstract] OR imbalance[Title/Abstract] OR coping[Title/Abstract] OR obligation[Title/Abstract] OR obligations[Title/Abstract] OR esteem[Title/Abstract] OR self-esteem[Title/Abstract] OR status control[Title/Abstract] OR money[Title/Abstract] OR cost[Title/Abstract] OR costs[Title/Abstract] OR gain[Title/Abstract] OR gains[Title/Abstract] OR break[Title/Abstract] OR breaks[Title/Abstract] OR job loss[Title/Abstract] OR piece rate work[Title/Abstract] OR role ambiguity[Title/Abstract] OR role-conflict*[Title/Abstract] OR work-role[Title/Abstract] OR working hour*[Title/Abstract] OR working time*[Title/Abstract] OR overtime[Title/Abstract] OR time-work[Title/Abstract] OR temporary work*[Title/Abstract] OR "occupational stress"[MeSH Terms] OR competition[Title/Abstract] OR "lifting"[MeSH Terms] OR "moving and lifting patients"[MeSH Terms] OR "physical exertion"[MeSH Terms] OR posture[Title/Abstract] OR postural[Title/Abstract] OR "walking"[MeSH Terms] OR "relaxation"[MeSH Terms] OR sitting[Title/Abstract] OR standing[Title/Abstract] OR sedentary[Title/Abstract] OR movement[Title/Abstract] OR "work schedule tolerance"[MeSH Terms] OR "personnel downsizing"[MeSH Terms] OR downsizing*[Title/Abstract] OR "organizational culture"[MeSH Terms] OR "bullying"[MeSH Terms] OR "workplace violence"[MeSH Terms] OR "social discrimination"[MeSH Terms] OR "prejudice"[MeSH Terms] OR "communication/psychology"[MeSH Terms] OR recuperation*[Title/Abstract] OR recovery[Title/Abstract] OR justice*[Title/Abstract] OR injustice*[Title/Abstract] OR boredom[Title/Abstract] OR boring[Title/Abstract] OR harassment[Title/Abstract] OR day-time[Title/Abstract] OR night-time[Title/Abstract] OR full-time[Title/Abstract] OR part-time[Title/Abstract] OR flexible work*[Title/Abstract] OR organizational change[Title/Abstract] OR organisational change[Title/Abstract] OR lean production[Title/Abstract] OR "Employee Performance Appraisal"[MeSH Terms] OR "Employee Grievances"[MeSH Terms] OR technostress[Title/Abstract] OR techno-stress[Title/Abstract] OR techno-stressor[Title/Abstract] OR techno-stressors[Title/Abstract] OR information system[Title/Abstract] OR information systems[Title/Abstract] OR techno-eustress[Title/Abstract] OR techno-distress[Title/Abstract] OR ubiquity[Title/Abstract] OR reliability[Title/Abstract] OR "ease of

	use”[Title/Abstract] OR presenteeism[Title/Abstract] OR interruption*[Title/Abstract] OR disruption*[Title/Abstract] OR distraction*[Title/Abstract] OR techno-insecurity[Title/Abstract] OR techno-overload[Title/Abstract] OR techno-invasion[Title/Abstract] OR techno-uncertainty[Title/Abstract] OR techno-complexity[Title/Abstract] OR technology[Title/Abstract] OR technologies[Title/Abstract] OR technological[Title/Abstract] OR technologization[Title/Abstract] OR technologisation[Title/Abstract] OR complexity[Title/Abstract] OR digital stress[Title/Abstract] OR computer stress[Title/Abstract] OR digital[Title/Abstract] OR digitalization[Title/Abstract] OR digitalisation[Title/Abstract] OR digitization[Title/Abstract] OR digitisation[Title/Abstract] OR information technolog*[Title/Abstract] OR IT[Title/Abstract] OR communication technolog*[Title/Abstract] OR information and communication technolog*[Title/Abstract] OR ICT[Title/Abstract] OR multi-tasking[Title/Abstract] OR information overload[Title/Abstract] OR “industry 4.0”[Title/Abstract] OR phone[Title/Abstract] OR phones[Title/Abstract] OR telephone[Title/Abstract] OR telephones[Title/Abstract] OR cellphone[Title/Abstract] OR cellphones[Title/Abstract] OR smartphone[Title/Abstract] OR smartphones[Title/Abstract] OR e-mail[Title/Abstract] OR tablet[Title/Abstract] OR tablets[Title/Abstract] OR wearable[Title/Abstract] OR wearables[Title/Abstract] OR smartwatch[Title/Abstract] OR smartwatches[Title/Abstract] OR mobile[Title/Abstract] OR mobility[Title/Abstract] OR device[Title/Abstract] OR devices[Title/Abstract] OR messaging system[Title/Abstract] OR instant messaging[Title/Abstract] OR messenger service[Title/Abstract] OR media[Title/Abstract] OR multimedia[Title/Abstract] OR automation[Title/Abstract] OR automatization[Title/Abstract] OR automatisation[Title/Abstract] OR computer[Title/Abstract] OR computers[Title/Abstract] OR computer-assisted[Title/Abstract] OR computer-based[Title/Abstract] OR computer-controlled[Title/Abstract] OR computer-driven[Title/Abstract] OR computerized[Title/Abstract] OR computerised[Title/Abstract] OR computer-navigated[Title/Abstract] OR computer-supported[Title/Abstract] OR computer-system[Title/Abstract] OR computer-program[Title/Abstract] OR computer-programme[Title/Abstract] OR computer-mediated[Title/Abstract] OR autonomous[Title/Abstract] OR machine[Title/Abstract] OR machines[Title/Abstract] OR internet[Title/Abstract] OR hyperconnectivity[Title/Abstract] OR speed[Title/Abstract] OR human-machine interaction*[Title/Abstract] OR human-robot interaction*[Title/Abstract] OR human-computer interaction*[Title/Abstract] OR artificial intelligence[Title/Abstract] OR robot*[Title/Abstract] OR techno-unreliability[Title/Abstract] OR work-life-blending[Title/Abstract] OR work-life-conflict*[Title/Abstract] OR work-life-balance[Title/Abstract] OR hardware[Title/Abstract] OR software[Title/Abstract] OR virtual reality[Title/Abstract] OR virtual communication[Title/Abstract] OR work intensity[Title/Abstract] OR performance monitoring[Title/Abstract] OR surveillance[Title/Abstract] OR performance assessment[Title/Abstract] OR performance appraisal[Title/Abstract] OR performance control[Title/Abstract] OR transparency[Title/Abstract] OR privacy[Title/Abstract] OR sense of achievement[Title/Abstract] OR non-availability[Title/Abstract] OR augmented reality[Title/Abstract] OR mixed reality[Title/Abstract] OR program[Title/Abstract] OR programme[Title/Abstract] OR predictability[Title/Abstract]

	**Outcome**
AND	“inflammation”[MeSH Terms] OR inflammat*[tw] OR “immune system”[MeSH Terms] OR “immune system phenomena”[MeSH Terms] OR immune[tw] OR immunology[tw] OR immunity[tw] OR "leukocytes"[MeSH Terms] OR leukocyte*[tw] OR eosinophil*[tw] OR granulocyte*[tw] OR lymphocyte*[tw] OR macrophage*[tw] OR monocyte*[tw] OR neutrophil*[tw] OR dendritic cell*[tw] OR “acute-phase proteins”[MeSH Terms] OR ("acute-phase"[tw] AND "proteins"[tw]) OR "acute-phase proteins"[tw] OR ("acute"[tw] AND "phase"[tw] AND "protein"[tw]) OR "acute phase protein"[tw] OR “c-reactive protein”[MeSH Terms] OR (“c-reactive”[tw] AND “protein”[tw]) OR “c-reactive protein”[tw] OR “c reactive protein”[tw] OR CRP[tw] OR “fibrinogen”[MeSH Terms] OR fibrinogen*[tw] OR “serum amyloid a protein”[MeSH Terms] OR "serum amyloid a protein"[tw] OR "serum amyloid a"[tw] OR “cytokines”[MeSH Terms] OR cytokine*[tw] OR “chemokines”[MeSH Terms] OR chemokine*[tw] OR “interferon-gamma”[MeSH Terms] OR interferon*[tw] OR IFN[tw] OR “interleukins”[MeSH Terms] OR interleukin*[tw] OR IL[tw] OR “lymphokines”[MeSH Terms] OR lymphokine*[tw] OR “monokines”[MeSH Terms] OR monokine*[tw] OR "tumour necrosis factor"[tw] OR "tumor necrosis factor-alpha"[MeSH Terms] OR ("tumor"[tw] AND "necrosis"[tw] AND "factor-alpha"[tw]) OR "tumor necrosis factor-alpha"[tw] OR ("tumor"[tw] AND "necrosis"[tw] AND "factor"[tw]) OR tumor necrosis factor*[tw] OR TNF[tw] OR "cell-free nucleic acids"[MeSH Terms] OR ("cell-free"[tw] AND "nucleic"[tw] AND "acids"[tw]) OR "cell-free nucleic acids"[tw] OR ("cell"[tw] AND "free"[tw] AND "dna"[tw]) OR "cell free dna"[tw] OR "inflammasomes"[MeSH Terms] OR inflammasome*[tw] OR "intercellular adhesion molecule-1"[MeSH Terms] OR ("intercellular"[tw] AND "adhesion"[tw] AND "molecule-1"[tw]) OR "intercellular adhesion molecule-1"[tw] OR "intercellular adhesion molecule 1"[tw] OR "transcription factor ap-1"[MeSH Terms] OR ("transcription"[tw] AND "factor"[tw] AND "ap-1"[tw]) OR "transcription factor ap-1"[tw] OR "activator protein 1"[tw] OR NF-IL6[tw] OR NF IL6[tw] OR “NF-kappa B”[MeSH Terms] OR “NF-kappa B”[tw] OR "NF kappa B"[tw]
	**Study design**
AND	longitudinal[Title/Abstract] OR prospective[Title/Abstract] OR follow-up[Title/Abstract] OR retrospective[Title/Abstract] OR intervent*[Title/Abstract] OR before-after[Title/Abstract] OR before-and-after[Title/Abstract] OR "interrupted time series analysis"[Title/Abstract] OR ("interrupted"[Title/Abstract] AND "time"[Title/Abstract] AND "series"[Title/Abstract] AND "analysis"[Title/Abstract]) OR ("interrupted"[Title/Abstract] AND "time"[Title/Abstract] AND "series"[Title/Abstract]) OR "interrupted time series"[Title/Abstract] OR "risk assessment"[MeSH Terms] OR ("risk"[Title/Abstract] AND "assessment"[Title/Abstract]) OR "risk assessment"[Title/Abstract]
